# FoxP3+ Regulatory T Cells Determine Disease Severity in Rodent Models of Inflammatory Neuropathies

**DOI:** 10.1371/journal.pone.0108756

**Published:** 2014-10-06

**Authors:** Gerd Meyer zu Hörste, Steffen Cordes, Anne K. Mausberg, Alla L. Zozulya, Carsten Wessig, Tim Sparwasser, Christian Mathys, Heinz Wiendl, Hans-Peter Hartung, Bernd C. Kieseier

**Affiliations:** 1 Department of Neurology, Heinrich-Heine-University, Medical Faculty, Düsseldorf, Germany; 2 Department of Neurology, Julius-Maximilians-University, Würzburg, Germany; 3 Institute for Infection Immunology, TWINCORE, Center for Experimental and Clinical Infection Research, Hannover, Germany; 4 Department of Diagnostic and Interventional Radiology, Heinrich-Heine-University, Medical Faculty, Düsseldorf, Germany; 5 Department of Neurology, University of Münster, Münster, Germany; Friedrich-Alexander University Erlangen, Germany

## Abstract

Inflammatory neuropathies represent disabling human autoimmune disorders with considerable disease variability. Animal models provide insights into defined aspects of their disease pathogenesis. Forkhead box P3 (FoxP3)+ regulatory T lymphocytes (Treg) are anti-inflammatory cells that maintain immune tolerance and counteract tissue damage in a variety of immune-mediated disorders. Dysfunction or a reduced frequency of Tregs have been associated with different human autoimmune disorders. We here analyzed the functional relevance of Tregs in determining disease manifestation and severity in murine models of autoimmune neuropathies. We took advantage of the DEREG mouse system allowing depletion of Treg with high specificity as well as anti-CD25 directed antibodies to deplete Tregs in mice in actively induced experimental autoimmune neuritis (EAN). Furthermore antibody-depletion was performed in an adoptive transfer model of chronic neuritis. Early Treg depletion increased clinical EAN severity both in active and adoptive transfer chronic neuritis. This was accompanied by increased proliferation of myelin specific T cells and histological signs of peripheral nerve inflammation. Late stage Treg depletion after initial disease manifestation however did not exacerbate inflammatory neuropathy symptoms further. We conclude that Tregs determine disease severity in experimental autoimmune neuropathies during the initial priming phase, but have no major disease modifying function after disease manifestation. Potential future therapeutic approaches targeting Tregs should thus be performed early in inflammatory neuropathies.

## Introduction

Inflammatory polyneuropathies constitute disabling disorders of the peripheral nervous system (PNS) including acute and chronic variants. The acute Guillain-Barré syndrome (GBS) features rapid onset, monophasic PNS inflammation [1], [2]. Experimental autoimmune neuritis (EAN) induced with myelin protein peptides serves as an animal model of the demyelinating GBS variant [3]. Animal models for the axonal variants have been generated by immunization against ganglioside components of the axonal membrane [4]. Chronic inflammatory demyelinating polyradiculo-neuropathy (CIDP) – the most common chronic inflammatory neuropathy – presents with chronic progressive or relapsing remitting sensory and motor impairments and features immune cell infiltration of peripheral nerves [5]–[7]. Animal models replicating the clinical and pathological hallmarks of chronic inflammatory neuropathies are less well established compared to models of acute neuritis. Mice of the autoimmune-prone non obese diabetic (NOD) strain with deficiency in the costimulatory molecules B7-2 [8] and intercellular adhesion molecule (ICAM)-1 [9] spontaneously develop chronic inflammation and demyelination of peripheral nerves and constitute potential animal models of CIDP. Both GBS and CIDP feature considerable disease variability and factors determining the severity and course of inflammatory neuropathies remain unknown. Specifically, it is poorly understood why PNS auto-inflammation remains acute monophasic in some, but progresses chronically in other patients.

Regulatory T cells (Tregs) expressing the transcription factor forkhead box protein 3 (FoxP3) are a naturally occuring anti-inflammatory T cell subset that is indispensable for the maintenance of self tolerance and immune homeostasis [10], [11]. Lack of functional Tregs causes fatal autoimmune diseases both in mice and humans [12], [13]. Furthermore, Treg dysfunction has been associated with different human autoimmune disorders [10]. Most FoxP3+ Tregs are CD4+ T cells expressing the interleukin-2 (IL-2) receptor α-chain (CD25) at high levels. They can suppress the activation, proliferation and effector functions of various effector immune cells. Reduced levels or an impaired function of Tregs have been described in human patients with GBS [14]–[16] and CIDP [17], [18]. In corresponding animal models, Tregs are present in the PNS during EAN [19] at reduced numbers [20] while their number increases with treatments ameliorating EAN [21]–[23]. Apart from these descriptive data, it remains unknown whether Tregs exert a functional role in the manifestation of inflammatory neuropathies. We here demonstrate that Tregs determine the disease severity and variability, but do not influence the conversion to chronicity in rodent models of acute and chronic inflammatory neuropathies.

## Material and Methods

### Active EAN in DEREG mice

Generation of mice expressing the human diphtheria toxin (DTx) receptor under the control of the FoxP3 promotor (DEREG mice) has been previously described [24]. The line was maintained in heterozygous breeding at a conventional animal housing facility. Animal experimentation was approved by the responsible state authorities. Genotyping was performed by routine PCR from tail biopsy DNA as described [24]. Active EAN was induced by subcutaneous injection of 200 µg myelin protein zero peptide spanning amino acids 180–199 (P0_180–199_) (JPT peptide technologies) emulsified in 100 µl complete Freund's adjuvant (CFA) (Difco) containing 1 mg/ml heat inactivated Mycobacterium tuberculosis strain H37RA mixed with 100 µl PBS into the flanks. Animals received intraperitoneal injections of 500 ng pertussis toxin (PTx) (Sigma-Aldrich) dissolved in 100 µl sterile phosphate buffered saline (PBS) on the day of immunization (day 0) and on day 2 after immunization (day 2). To achieve Treg depletion, DTx (Merck) was dissolved in 100 µl sterile PBS and applied intraperitoneally on d3 and d4 at 50 µg/kg (1 µg per mouse). Two independent experiments were performed including 4–6 animals per group respectively. A modified clinical EAN score [25] was applied daily by a blinded investigator (A.Z.) to quantify impairments: 0 no impairments, 1 reduced tone of the tail, 2 limp tail, 3 absent righting reflex or clasping of the hind limbs, 4 gait ataxia, 5 mild paraparesis, 6 moderate paraparesis, 7 severe paraparesis or paraplegia, 8 tetraparesis, 9 moribund, 10 death due to neuropathy. At the end of the observation period electrophysiology was performed and animals were sacrificed by cervical dislocation. Flow cytometry was performed from purified splenocytes.

### Active EAN in SJL mice

Animals were maintained at the central animal facility of the Heinrich-Heine-University, Düsseldorf under specific pathogen free conditions and transferred to conventional housing for experimentation. Animal experimentation was approved by the responsible state authorities (Landesamt für Natur, Umwelt und. Verbraucherschutz Nordrhein-Westfalen) with the approval reference numbers 8.87–50.10.34.08.207 and 50.05-230-65/06. Active EAN was induced by subcutaneous injection of 2,5 mg lyophilized bovine peripheral nerve myelin (bPNM) as previously described [26] emulsified in 200 µl CFA/PBS mixture together with PTx injections on day 0 and 2 after immunization as described above. Phenotyping was performed every second day by a blinded investigator (S.C.) using the clinical EAN score described above and hind limb grip strength measurement (see below). For early Treg depletion, mice received intraperitoneal injections of purified anti-mouse CD25 antibody (no azide, low endotoxin, clone PC61, rat IgG1, BioLegend) at 0.125 mg/mouse/day on day 4 to day 1 before immunization (day -4 to day -1). This clone had been previously demonstrated to deplete Tregs [27]. Late Treg depletion was performed by antibody injection on days 14 to 17 after immunization. Control mice received comparable amounts of isotype control antibody (rat IgG1, BioLegend). Blood was taken from experimental animals by tail vein bleeding 4 days before and 4, 10, 14, 22 and 30 days after immunization and the percentage of FoxP3+ cells was determined by flow cytometry. Electrophysiogy was performed at the end of the experiment 28 days after immunization. Two independent experiments were performed with 5–6 animals per group respectively.

### Adoptive transfer neuritis in NOD-SCID mice

ICAM-1^-/-^ NOD mice spontaneously develop a chronic inflammatory neuropathy that can be adoptively transferred to immunodeficient hosts by isolated CD4+ T cells [28]. Splenocytes were purified from approximately one year old, clinically affected ICAM-1^-/-^ NOD mice. After 48h in culture, cells from all donor animals were pooled, washed three times with PBS, adjusted to 23×10^6^ cells suspended in 400 µl PBS per animal and intravenously injected into 6 to 8 weeks old host immunodeficient severe combined immunodeficient (SCID) mice on NOD background (Taconic) subsequently named NOD-SCID mice. Recipient mice were maintained under specific pathogen free conditions for up to 10 weeks and were analyzed for phenotypic signs of neuropathy twice per week by a blinded investigator (S.C.). Treg depletion was performed by intraperitoneal antibody application as described above on days 6 to 10 (early depletion) and days 37 to 40 (late depletion) after adoptive transfer. Electrophysiogy was performed at the end of the experiment 50 and 70 days after transfer in early and late depleted animals, respectively.

### Grip strength analysis

The standardized grip strength test was performed for hind limbs as previously described [29]. Briefly, mice were grasped at the proximal base of their tail and pulled towards a horizontal T-bar (width 12 cm, diameter 1.5 mm) connected to a gauge with increasing force. The maximum force (measured in Newton) exerted onto the T-bar before the animals lost grip was recorded. Four measurements were performed at each observation day and average values were calculated. To reduce the impact of learning and training on the test results, grip strength was measured at days 14, 13, 12 and 6 before immunization and the relative change in hind limb grip strength compared to average pre-immunization values was calculated for further analysis.

### Electrophysiology

Mouse sciatic nerve conduction properties were determined as previously described [29]. Briefly, mice were anaesthetized using intraperitoneal injection of ketamin (100 mg/kg) and xylazin (5 mg/kg), while constant body temperature was maintained using a heating plate connected to a rectal temperature sensor. Two recording electrodes were inserted into the small foot muscles to assess motor response. Two monopolar stimulating electrodes were placed dorsal of the ankle and at the sciatic notch enclosing the sciatic nerve for distal and proximal stimulation, respectively. Stimulation was performed with increasing current until supramaximal stimulation was achieved. Maximum compound muscle action potential (CMAP) amplitude voltage (mV) was recorded. Nerve conduction velocity (NCV, m/s) was calculated as the quotient between the distance and the difference of motor latencies between proximal and distal stimulation. Average values were calculated from two independent recordings per animal.

### Histology

Animals were sacrificed by cervical dislocation and directly intracardially perfused with phosphate buffered saline (PBS) followed by 4% paraformaldehyde. Sciatic nerves were dissected, paraffin embedded, cut into 7 µm sections on a standard microtome and haematoxylin-eosin (HE) stained following standard protocols. For immunohistochemical staining, nerve sections were incubated with anti-mouse CD3 antibody (ab5690, Abcam) followed by a biotinylated goat anti-rabbit secondary antibody (BA-1000, Vector) and a streptavidin-biotin-horseradish peroxidase complex (DAKO). 3,3′-diaminobenzidine (DAB) was added as peroxidase substrate according to manufacturer's instructions. Between all protocol steps sections were washed for five minutes in PBS. Slides were dehydrated and mounted in xylene based medium (Merck). All incubations were performed at room temperature. The entire sciatic nerve section was photographed using a standard microscope (Zeiss) and photographs were photomerged using Photohop CS3 (Adobe). The total endoneural area was measured and the number of CD3 reactive cell nuclei was counted on merged photographs of the sections by a blinded investigator (S.C.) using the CellCounter plug-in of ImageJ (v143, NIH). The density of CD3+ cells per mm^2^ section area was calculated.

### Cell preparation and flow cytometry

Splenocytes were extracted from dissected spleens by passing through a 40 µm cell strainer followed by erythrocyte lysis (both BD Biosciences). Splenocytes were either cultured (1×10^7^/well) in 6-well plates stimulated using soluble antibodies against CD3 (1 µg/ml, 145-2C11, from BD Pharmingen) and CD28 (0,5 µg/ml [PV-1] from Abcam) at 37°C in a humidified CO_2_ incubator for adoptive transfer, proliferation experiments or stained for flow cytometry. Blood taken by tail vein bleeding was collected in 75 µl hematocrit capillaries (Radiometer Clinical Aps) and transferred into 1 ml of PBS/1%FCS/10 mM EDTA solution followed by erythrocyte lysis. Flow cytometry was performed from blood and spleen cells. Cells were stained for cell surface CD4 (L3T4, APC or Pacific Blue labelled), CD25 (3C7, PE labelled) and intracellular FoxP3 (MF23, Pacific Blue or APC labelled) using the FoxP3 staining buffer set (all from BD Biosciences). Flow cytometry was performed using a FACSCanto II flow cytometer (BD Biosciences). To assess the autoimmune proliferatory response after Treg depletion, splenocytes from NOD-SCID mice having previously received adoptive transfer of ICAM-1^-/-^ NOD lymphocytes and injections of anti-CD25 antibodies were cultured in the presence of syngenic mouse sciatic nerve homogenisate. 2×10^5^ splenocytes were maintained in 96-well plates for 96 hours. ^3^H-Thymidin (Hartmann Analytic) was added for the last 24 hours and proliferation was assessed in quadruplicate wells by measuring ^3^H-Thymidine incorporation. Stimulatory indices were calculated by dividing counts per minute (CPM) of each well by the average CPM of non-stimulated wells.

### Data acquisition and analysis

All flow cytometry data were analyzed using FlowJo software (v7.2.5 TreeStar). Data were statistically analyzed using GraphPadPrism 5.0 (GraphPad Software). The Wilcoxon-Mann-Whitney and Student's t-test for unrelated samples were used to test for statistically significant differences of non-Gaussian and Gaussian distributed data, respectively. Differences were considered significant at p-values <0.05.

## Results

### DTx mediated Treg depletion deteriorates EAN in DEREG mice

**Figure 1:**
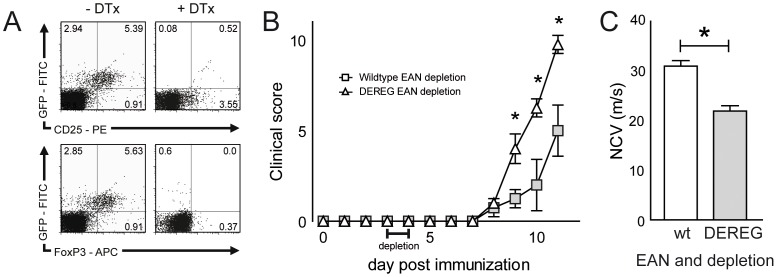
Treg depletion in DEREG mice exacerbates EAN. (A) DEREG mice expressing the human diphtheria toxin (DTx) receptor under the control of the FoxP3 promotor together with green fluorescent protein (GFP) reporter were injected with 50 µg/kg DTx (1 µg per mouse) on days 3 and 4 after immunization against myelin protein zero peptide P0_180–199_. Flow cytometry dot plots depict the CD4+ fraction of DEREG splenocytes stained against FoxP3 and CD25 7 days after intraperitoneal PBS (left panels) or DTx (right panels) injection. (B) DEREG (n = 4) and control (n = 4) mice were immunized with myelin protein peptide P0_180–199_ and received DTx injections on days 3 and 4 after immunization and daily clinical scoring was performed until day 11 post immunization. (C) Sciatic nerve conduction velocity was assessed on day 11 after EAN induction in diphtheria toxin treated wildtype (left bar) and DEREG mice (right bar). Plots depict mean ± SEM in panels B and C. One representative out of two independent experiments is depicted. DTx diphthteria toxin, wt wildtype, NCV nerve conduction velocity. * p<0.05.

We used different approaches to study the importance of Tregs in EAN. The previously described DEREG mouse line expresses the human DTx receptor under the control of the FoxP3 promotor together with a GFP reporter [24]. Expression of the human DTx receptor confers toxin sensitivity to othwerwise toxin resistant mouse cells and thus allows cell type specific Treg depletion by DTx [24], [30]. Intraperitoneal DTx application successfully depleted FoxP3+ cells from the periphery as traced by GFP expression and FoxP3 staining (Fig. 1A). DTx mediated Treg depletion after EAN induction significantly increased the severity of EAN symptoms (Fig. 1B). The average sciatic nerve conduction velocity was significantly reduced in Treg depleted DEREG mice compared to wildtype mice (Fig. 1C). As previously described, active immunization – which is required for EAN induction – triggers lethal toxicity of DTx independent of its Treg depleting effect already in conventional C57BL/6 mice [31]. In the current setting, clinical signs of EAN and DTx toxicity could not be safely distinguished, preventing further detailed analyses of Treg depletion during the course of EAN in DEREG mice.

### Antibody mediated Treg depletion deteriorates EAN in SJL mice

**Figure 2:**
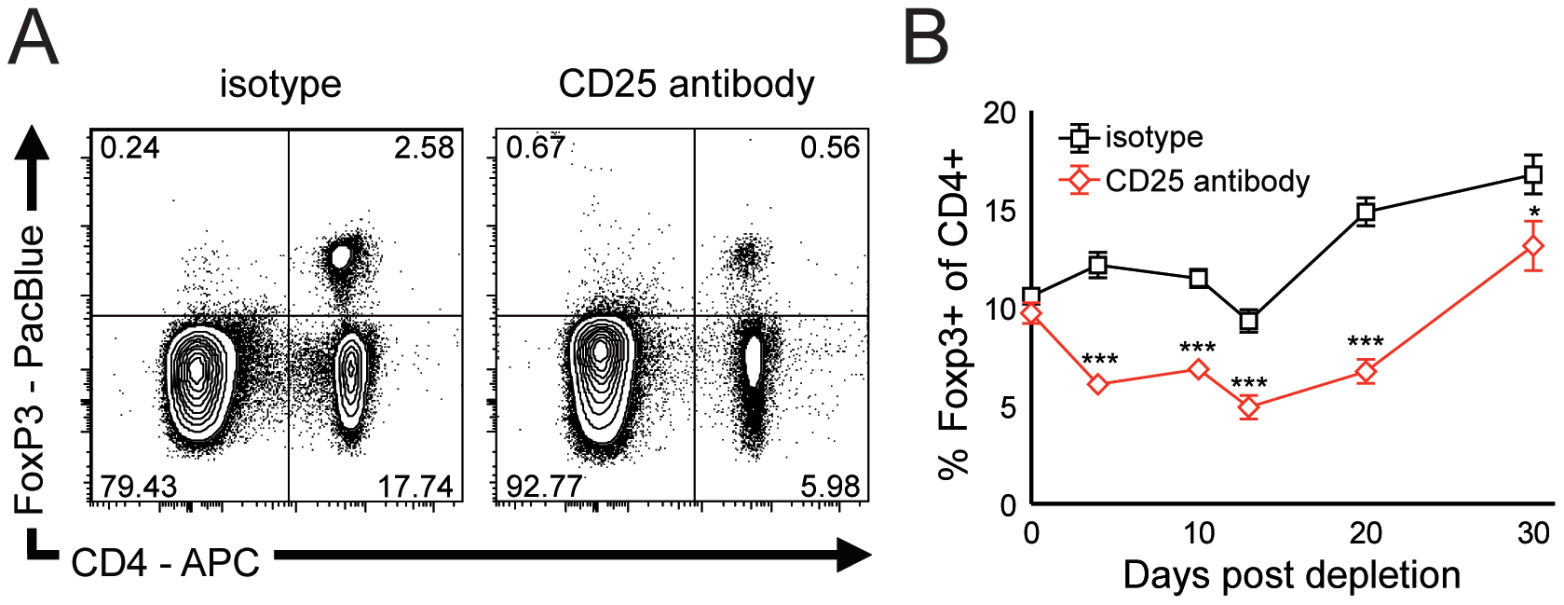
CD25 directed antibody continuously depletes Tregs. (A) SJL mice received intraperitoneal injections of CD25 directed antibody (clone PC61) or isotype control. Flow cytometry contour plots depict blood mononuclear cells from isotype (left panel) and CD25 antibody (right panel) treated animals stained for FoxP3 and CD4. (B) The percentage of FoxP3+ of blood CD4+ cells in isotype (black line) and CD25 antibody (red line) treated animals at 4, 10, 14, 22 and 30 days after depletion was determined by flow cytometry. *** p<0.005.

**Figure 3:**
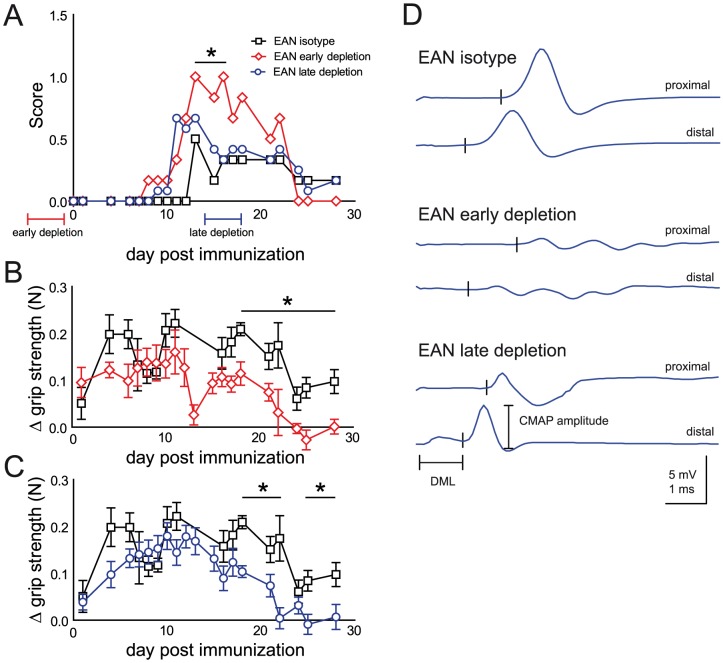
Treg depletion deteriorates active EAN in SJL mice. (A) Active EAN was induced by subcutaneous immunization with myelin protein zero peptide P0_180–199_ in complete Freund's adjuvant. Animals received intraperitoneal injections of CD25 antibody on days 4 to 1 before (early depletion) and day 14 to 17 (late depletion) after immunization. Animals were regularly checked for impairments and a clinical score was assigned in a blinded fashion. The mean score is depicted. (B) Hind limb grip strength was measured from day 14 before immunization onwards and the change (Δ) in grip strength measured in Newton (N) relative to average pre-immunization values was calculated for each individual day. Values of isotype treated controls compared to early and late depleted animals are depicted in the top and bottom panel, respectively. Plots depict mean ± SEM in panels B and C. One out of two independent experiments is shown. (D) Sciatic nerve electrophysiology was performed on day 28 after immunization and representative measurements of individual animals are displayed. Individual early depleted animals (middle panel) presented electrophysiological signs of demyelination such as prolonged distal motor latency (DML) and reduced compound muscle action potential (CMAP) amplitudes. * p<0.05.

**Figure 4:**
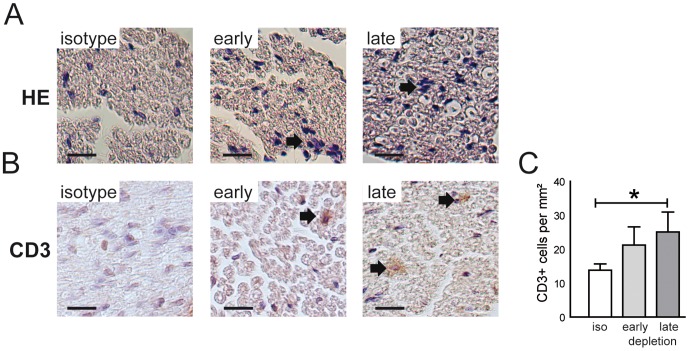
Treg depletion increases PNS T cell infiltration in active EAN. (A) Randomly chosen animals from all groups were sacrificed at day 15 post immunization and sciatic nerve paraffin sections stained with haematoxyllin-eosin demonstrates infiltration of mononuclear cells (arrows) in early (middle panel) and late (right panel) depleted animals. (B) Sciatic nerve sections were stained against CD3. Depleted animals had a higher frequency of CD3+ T cells. (C) The number of CD3+ T cells was manually counted and the relative cell density was quantified. The density of CD3+ cells was significantly higher in late depleted animals. Scale bars depict 50 µm in panels A and B. * p<0.05.

We therefore utilized anti-CD25 directed antibody mediated Treg depletion as previously described in models of central nervous system inflammatory demyelination [32]. Indeed, intraperitoneal applications of anti-CD25 antibody significantly reduced the percentage of FoxP3+ cells in blood cells of SJL mice (Fig. 2A). This strong reduction persisted for approximately three weeks until Treg numbers increased again (Fig. 2B). Immunization of SJL mice with bovine peripheral nerve myelin induced EAN with little impairments (Fig. 3A). Application of CD25 antibody before immunization (day -4 to -1) increased peak EAN severity (Fig. 3A). Antibody application at the peak of EAN severity (day 14 to 17) did not alter the clinical score (Fig. 3A). In addition to clinical scoring, we utilized hind limb grip strength as quantitative measure of impairments in EAN. In accordance with the clinical phenotype, hind limb grip strength decreased after EAN induction (Fig. 3B). Treg depletion significantly increased the strength loss associated with EAN induction, both when applied before immunization and at the peak of disease (Fig. 3C). Sciatic nerve conduction studies revealed a dispersion of compound muscle action potentials and reduced nerve conduction velocities in a proportion of animals having received early and late Treg depletion (Fig. 3D). No abnormalities were detected in control animals (Fig. 3D). Histology identified focal infiltration of mononuclear cells in the sciatic nerve of Treg depleted mice with EAN (Fig. 4A). CD3+ T cells could be observed at higher frequencies in depleted animals (Fig. 4B). The density of CD3+ T cells was increased in depleted animals and was highest in animals after late Treg depletion (Fig. 4C).

### Antibody mediated Treg depletion deteriorates adoptively transferred neuritis

**Figure 5:**
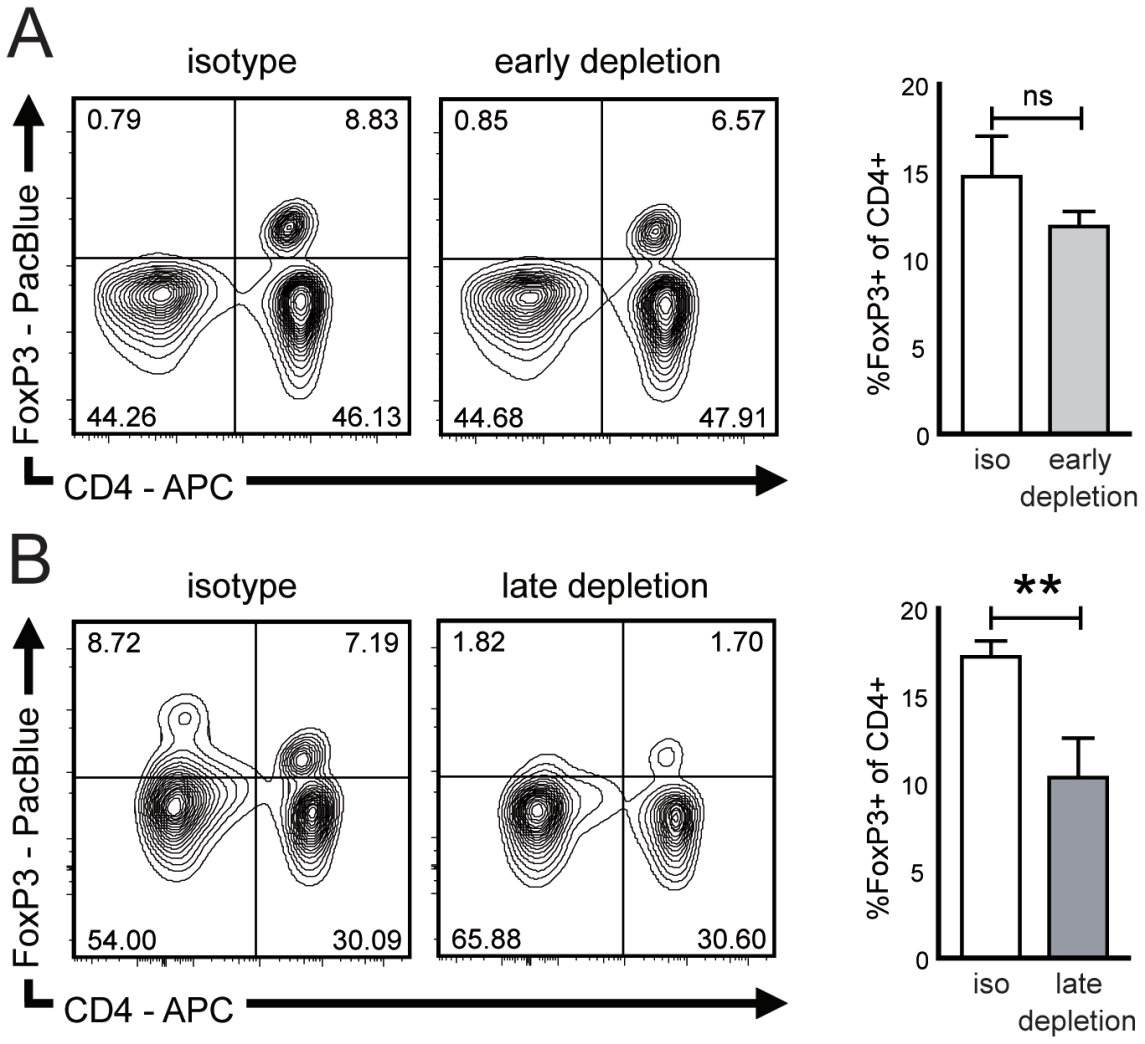
CD25 directed antibody depletes Tregs and increases myelin autoreactivity in adoptive transfer neuritis. (A) Lymphocytes from ICAM-1^-/-^ NOD mice were adoptively transferred into immunodeficient NOD-SCID mice. Recipients received intraperitoneal injections of isotype control (left panel) or CD25 antibody (middle panel) and the proportion of FoxP3+ spleen cells was assessed by flow cytometry. On day 50 after transfer – 40 days after early depletion – the percentage of FoxP3+ cells in early depleted animals showed a non-significant trend towards reduction (right panel). (B) On day 70 after transfer – 30 days after late depletion – the percentage of FoxP3+ cells was analyzed by flow cytometry and significantly reduced in late depleted animals. Box plots depict mean ± SEM in all panels. ns non significant, iso isotype control, ** p<0.01.

Murine EAN in our hands features low disease severity, making clinical assessment difficult in this model. Also, the spontaneous neuritis in ICAM-1 deficient (ICAM-1^-/-^) NOD mice is experimentally difficult to utilize due to its highly variable onset [28]. To circumvent these limitations, we adoptively transferred chronic neuritis from ICAM-1^-/-^ NOD mice into immunodeficient hosts thus triggering neuritis with synchronized onset but at the same time clinically resembling aspects of human chronic inflammatory neuropathies. Again application of the CD25 antibody reduced the percentage of FoxP3+ cells in NOD-SCID recipients. This reduction exhibited a non-significant trend in early depleted animals analyzed 40 days after depletion (Fig. 5A) and was significant in late depleted animals 30 days after depletion (Fig. 5B).

**Figure 6:**
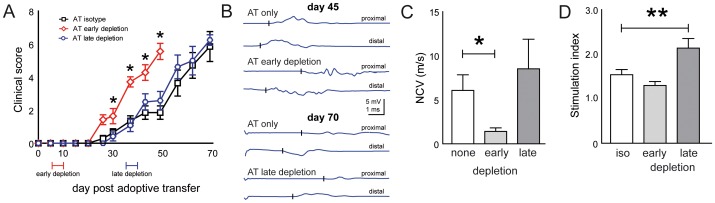
Treg depletion deteriorates adoptive transfer neuritis. (A) Following adoptive transfer of lymphocytes from ICAM-1^-/-^ NOD into immunodeficient NOD-SCID mice, recipients received intraperitoneal injections of CD25 antibody between day 7 and 10 (early depletion) or between day 37 and 40 (late depletion) after transfer. Clinical scoring was performed in a blinded fashion. (B) Sciatic nerve electrophysiology was performed when terminating the early (top panel) and late (lowel panel) depletion experiment at 45 and 70 days after transfer, respectively. (C) The average nerve conduction velocity (NCV) in all experimental groups was calclulated. Plots depict mean ± SEM in panels A and C. iso isotype control. (D) Lymphocytes extracted from adoptive transfer recipient mice were restimulated in culture with sciatic nerve homogenisates and T cell proliferation was measured by ^3^H-Thymidin incorporation in quadruplicate wells. Stimulation indices were calculated as multiple of unstimulated wells.

**Figure 7:**
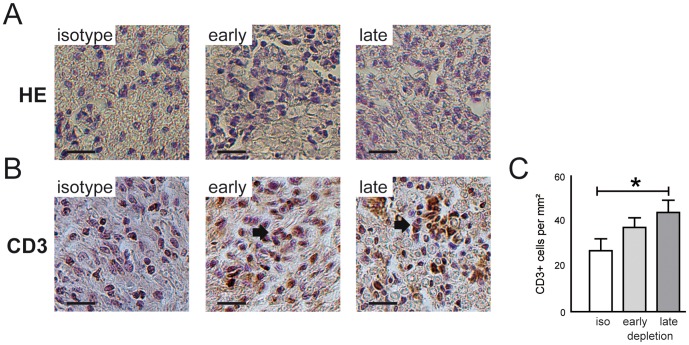
Treg depletion increases PNS T cell infiltration in adoptive transfer neuritis. (A) When terminating the experiment, sciatic nerve histology was generated. Paraffin sections stained with heamatoxyllin-eosin demonstrate vast infiltration of mononuclear cells in animals from all experimental groups. (B) Sciatic nerve sections were stained against CD3 and depleted animals had higher frequencies of CD3+ T cells. (C) The relative cell density of CD3+ T cells was quantified and was significantly higher in late depleted animals. Scale bars depict 50 µm in panels A and B. * p<0.05.

Adoptive transfer of ICAM-1^-/-^ NOD lymphocytes into NOD-SCID mice caused progressive impairments first manifesting 20 days after transfer (Fig. 6A). Early Treg depletion (days 7 to 10 after transfer) accelerated the manifestation of neuritis symptoms and shortened the latency until recipient animals reached moderate paraparesis (score 6) by 15 days in comparison to controls (Fig. 6A). Animal experimentation was then terminated for ethical reasons. Late depletion (days 37 to 40) did not alter the clinical course of the adoptively transferred neuritis (Fig. 6A). Electrophysiological abnormalities were more pronounced in early depleted animals (Fig. 6B) and average nerve conduction velocities were significantly lower in early depleted recipient mice (Fig. 6C). We have previously demonstrated that ICAM-1^-/-^ NOD mice show autoreactivity against components of their peripheral nerve myelin [28]. We here found, that lymphocytes from late depleted animals showed greater lymphocyte proliferation reactivity against sciatic nerve tissue than controls (Fig. 6D). In sciatic nerve paraffin sections the density of cell nuclei did not differ between depleted and non-depleted adoptive transfer recipients (Fig. 7A). The frequency of CD3+ T cells was higher in depleted mice than in controls (Fig. 7B). Quantification found an increased density of CD3+ T cells after late Treg depletion (Fig. 7C).

## Discussion

Tregs are indispensable for the maintenance of self tolerance and immune homeostasis and Treg dysfunction has been associated with various human autoimmune disorders and has been discussed as one potential factor driving disease progression. Reduced levels or an impaired function of Tregs have been described in human patients with acute and chronic inflammatory neuropathies. One study reported a transiently reduced number of FoxP3^+^ Tregs during acute stages of GBS while their suppressive function was unaltered [16]. Two technically less stringent studies reported a reduction in CD4^+^CD25^+^ T cells, which may include activated T effector cells in the acute phase of GBS without studying FoxP3 expression [14], [15]. In CIDP patients, one study found a reduction in the proportion and suppressive function of FoxP3^+^ Tregs [17], while another study reported an unchanged proportion, but decreased suppressive function [33]. In animal models, Tregs have been shown to be present in the PNS during acute stage EAN [19] and that their numbers are reduced during EAN [20]. Several studies reported an increase in Treg numbers with treatments ameliorating EAN [21]–[23].

Previous studies have not addressed, however, if Tregs functionally determine the manifestation of inflammatory neuropathies and if Treg numbers may represent a factor determining chronicity of autoimmunity in the peripheral nerve. We here demonstrate that depletion of Tregs increases the disease severity of actively induced and adoptively transferred murine autoimmune neuritis models. We conclude that Tregs suppress autoinflammatory reactions in the PNS and determine disease severity in inflammatory neuropathies. In synopsis with the previously published descriptive studies in human neuritis patients and corresponding animal models our findings are thus the first to demonstrate a functional relevance of Tregs in peripheral neuritis.

Depletion had more pronounced effects if performed early in the course of the respective disease model, indicating that Tregs constrain inflammation during the priming phase of EAN and during the early phases of adoptively transferred neuritis. Although Tregs are required to prevent autoimmunity throughout the lifespan of mice [30], reducing their numbers does not increase the severity of neuritis if disease has already manifested. Our findings thus do not support the hypothesis that Tregs determine whether autoimmunity in the PNS remains acute and monophasic as in GBS or chonically progressive as in CIDP. There are no data available to determine whether Tregs take effect in the periphery or locally in the PNS but our data may indicate that the early priming phase in the periphery is the more important site of action for Tregs in inflammatory neuropathies.

Our analysis of Tregs was impeded by several methodical difficulties. Firstly, when establishing the DEREG mouse model, we found that DTx could not be combined with active immunization of any kind due to a non-cell type specific probably septic reaction [31]. Therefore, analyses in this model had to remain limited. The model especially did not allow to differentiate between toxic and paretic symptoms making clinical scoring inadaequate. This is why we used CD25 directed antibodies for the depletion of Tregs. This is – naturally – less specific, but has provided crucial insights in the pathophysiological relevance of Treg previously. In accordance with previous studies in experimental autoimmune encephalomyelitis (EAE) [27], [32] we could still demonstrate successful and prolonged reduction of Treg numbers following CD25 antibody treatment supporting the adeaquacy of our approach.

Initially EAN was established in different non-murine species and preferentially used in the Lewis rat strain [34]. More recently, EAN induction has been described in different mouse strains [35]–[37]. In contrast to these published studies, however, we and others (Ralf Linker, University of Erlangen, personal communication) have repeatedly failed to induce severe and thus clinically well traceable EAN in mice. We used a bovine peripheral nerve myelin induced EAN in SJL mice, which in our experience features the greatest disease severity and reliability [36]. We further attempted to circumvent the low disease severity by using quantitative hind limb grip strength assessment as improved measure of paresis. Referential values in comparison to pre-immunization grip strength allowed to exclude learning and training effects. Here, we found an increased EAN severity following Treg depletion. Still, the effect of Treg depletion was less pronounced than expected. Previous investigations on the role of Treg were mainly performed by using depleting anti-CD25 antibodies. As a limitation, one has to consider that CD25 is also up-regulated on activated T cells [38] and there is evidence for a CD25-negative Treg subpopulation, which is not depleted [39].

We additionally used an adoptive transfer paradigm to elicit a severe progressive autoimmune neuropathy previously described in ICAM-1^-/-^ NOD mice [28]. Using a defined donor cell pool, we ensured that all animals received equal amounts of neuritogenic cells and that the resulting neuritis thus features a defined onset and severity. Early, but again not late, Treg depletion increased disease severity in this transfer model. This contrasts with the finding that Treg numbers were not reduced and PNS myelin directed autoreactivity was not increased in early depleted animals. The interval between depletion and flow cytometric and proliferation analysis was 10 days greater in early than in late depleted animals explaining the discrepancy. Of note, myelin auto-reactivity increased in late depleted animals, but was insufficient to further deteriorate the developing neuropathy. One may speculate that the speed of development of neuropathy cannot accelerate further after its onset. It remains to be determined if Tregs could actively inhibit neuritis if induced at this time point.

In conclusion, we have demonstrated the functional relevance of Tregs for the manifestation and severity of inflammatory neuropathies. In synopsis with previous descriptive human data, this identifies Tregs as potential therapeutic target in the early stage of disabling inflammatory neuropathies.
